# Nonneoplastic Tongue Swellings of Lymphatic and Lymphocytic Origin: Three Case Reports

**DOI:** 10.1155/2016/3180239

**Published:** 2016-11-20

**Authors:** Manar A. Abdul Aziz, Nermin M. Yussif

**Affiliations:** ^1^Oral Pathology Department, Faculty of Oral & Dental Medicine, Cairo University, Cairo, Egypt; ^2^National Institute of Laser Enhanced Sciences (NILES), Cairo University, Cairo, Egypt

## Abstract

Tongue is formed of a mass of muscles and salivary gland embedded in anterior highly vascular and posterior lymphoid stroma and covered by specialized surface epithelium. Growths from all of these heterogonous components may occur resulting in a wide variation in clinical features and behavior, ranging from self-limiting to aggressive lesions. Therefore, surgical excision is the treatment of choice. The aim of the current study is to report three different lesions that came to the Oral Surgery Department in the Faculty of Oral and Dental Medicine, Cairo University. Following clinical and histopathological examination, the diagnosis of reactive lymphoproliferative lesion, cystic lymphoepithelial lesion, and developmental lymphatic vessel malformation was reached.

## 1. Introduction

Tongue is a unique vital structure that favors great importance to the oral health. It usually appears at the fourth week of fetus intrauterine life. The anterior two-thirds of the tongue is formed by the fusion of both primary pharyngeal arches and tuberculum impar. While the posterior one-third arises from the 2nd, 3rd, and 4th pharyngeal arches [1].

Tongue is a highly vascular and muscular organ that is involved in a mucous sac [1]. This sac is smooth in relation to the ventral surface of the tongue and rough containing variant types of lingual papillae in relation to the dorsal surface. The ventral surface is more vascular than the dorsal one with thin webbed projections (plica fimbriata) arising within the lingual frenum while the dorsal surface is divided by groove into papillary anterior two-thirds and lymphoid posterior one-third. Lymphoid tissue is found in the posterior one-third of the tongue. It is a part of Waldeyer ring [2].

Its nomenclature as a muscular hydrostat arises from its ability to move and to give skeletal support for this motion. These hydrostatic properties are related to the specialized muscle arrays and muscle fibers. Therefore, tongue controls critical function such as speech, taste sensation, swallowing, and cleansing of the oral cavity. Tongue provides clue and reflection about the systemic condition. If lingual deformation or pathology occurs, these functions become impaired [1]. This developing pathology differs from congenital abnormalities and idiopathic lesions into infections and carcinogenic disorders. Tongue lesions may be short-term or long-term lesions which are classified according to their location, nature, composition, depth, and behavior. They are commonly classified into developmental, reactive, benign, or malignant lesions. The color and consistency differ from one lesion to another (Table 1). Recognition and diagnosis of these lesions require adequate knowledge about the basic anatomy of the tongue, comprehensive examination, and proper history. As a rule, most of the tongue lesions resolve fast as regards the high blood supply unless they have to be biopsied to exclude the malignancies [3, 4].

Lymphangiomas are congenital malformations that are derived from the lymphatic vessels. They are mainly related to the head and neck. There are various theories that explain their origin. They are mainly formed due to defect occurring during embryogenesis. They can be capillary, cavernous, or cystic [6]. Intraoral lymphangiomas are rare and arise mainly in the buccal mucosa, lips, palate, and lateral and posterior surface of the tongue. The main problem caused by such lesions is macroglossia that causes limitation of tongue movement, bleeding on trauma, and sleeping apnea. They may be superficial or deep. Superficial lesions usually appear as a pebbly surface which may affect the surface texture and color. On the other hand, the deep lesions do not exhibit change in the tongue surface or color but cause diffuse enlargement. Depending on their location, size, depth, and accessibility, they can be removed using conventional blade technique, cryotherapy, or laser. Sclerotherapy can also be used with inaccessible lesions [7].

Lymphoepithelial cyst is rare idiopathic intraoral condition. It usually arises in relation to the lateral and ventral surface of the tongue [8, 9]. Although palate is not a common location of this type of cysts, it may arise in relation to palatine tonsils and hard and soft palate. There is no age or sex predilection. It is painless lesion unless traumatized. Its treatment is restricted to the surgical excision [10–12].

Lymphoid hyperplasia is one of the rare lymphoproliferative lesions. Although it has common clinical and histopathological features with oral carcinoma, it is benign in nature. It usually appears as painless malignant-like ulcer [13, 14].

## 2. Case  1

A fifty-eight-year-old female patient came to the Department of Oral and Maxillofacial Surgery, Faculty of Dental Medicine, Cairo University, complaining of a swelling in the right side of the tongue. The presence of this swelling caused also difficulty in speech and deglutition.

**Figure 1:**
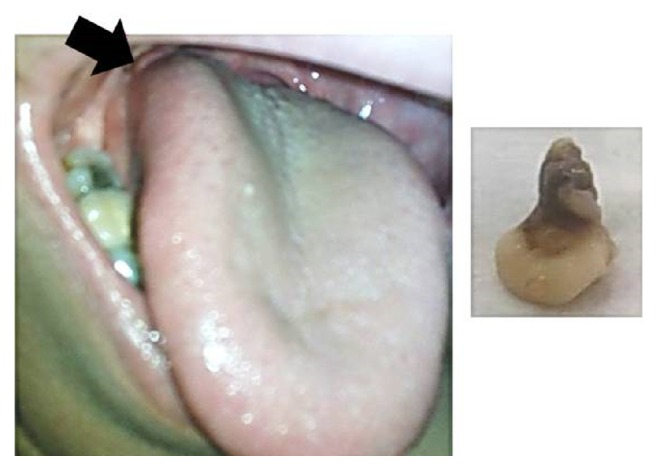
Clinical aspect of lymphoepithelial cyst in relation to the right lateral tongue border and macroscopic picture of the submitted biopsy.

Upon clinical examination, a well-circumscribed pinkish white nodule approximately 1 × 1 cm in size and round in shape was noted on the right lateral border of posterior third of the tongue (Figure 1). The surface was smooth. Upon palpation, it was soft and nontender.

As a differential diagnosis, fibroma, lymphoid hyperplasia, lymphoepithelial cyst, choristoma, lymphoma, and salivary gland neoplasm were included.

The excisional biopsy was performed at the Oral Surgery Department and the surgical specimen was submitted for microscopic examination in the Department of Oral and Maxillofacial Pathology, Faculty of Dental Medicine, Cairo University. In macroscopic examination, the specimen was received as one mass that appeared rounded in shape of about 1.0 × 1.2 cm in size with a narrow stump of about 1.0 × 0.6 cm. The specimen was whitish in color and soft in consistency (Figure 1).

**Figure 2:**
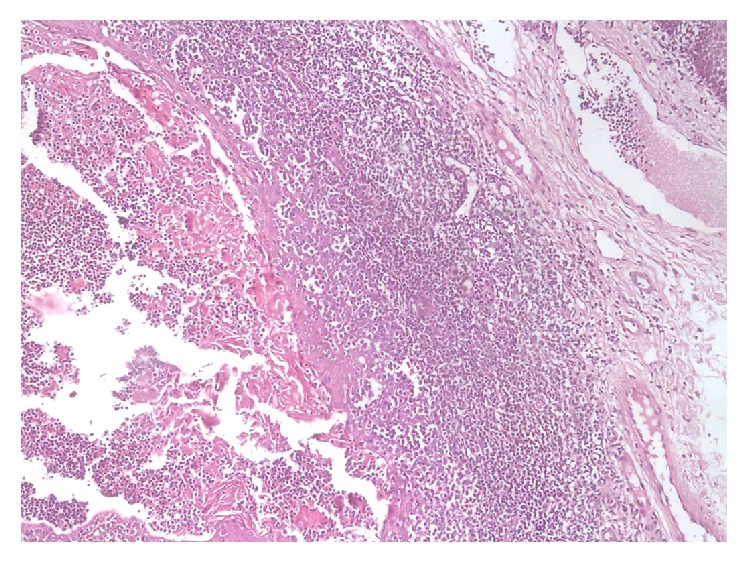
Photomicrograph of lymphoepithelial cyst showing cystic cavity lined by keratinized epithelium and filled with desquamated keratin intermixed with lymphocytes (H&E ×100).

Histopathological examination revealed a cystic cavity lined by orthokeratinized stratified squamous epithelium with uneven thickness. Within the lumen, keratin sloughs interspersed with lymphocytes were observed. The connective tissue wall contained well-demarcated aggregates of lymphocytes. Few germinal centers were detected within the lymphoid tissue. The lesion was covered by keratinized stratified squamous epithelium (Figure 2). Immunohistochemical reaction to CD3 and CD20 showed normal appearance of germinal center excluding the malignant nature of the lesion. The final diagnosis was designated as an oral lymphoepithelial cyst.

## 3. Case  2

A sixty-year-old female patient came to the Department of Oral and Maxillofacial Surgery, Faculty of Dental Medicine, Cairo University, complaining of bilateral swellings in the tongue. The patient noticed the enlargement of the left side mass.

**Figure 3:**
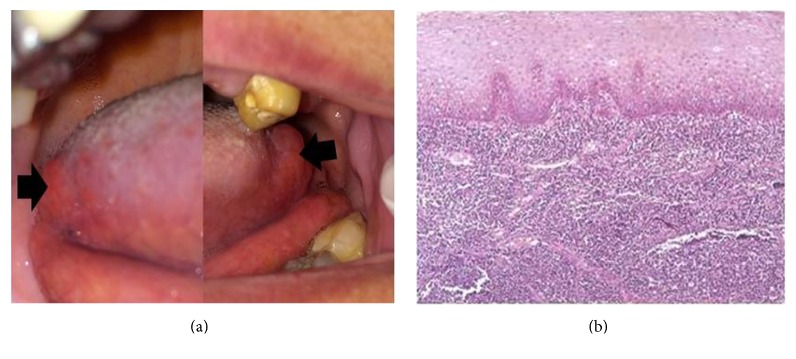
(a) Clinical aspect of bilateral lymphoid hyperplasia in relation to the posterior part of lateral border of the tongue. (b) Photomicrograph of lymphoid hyperplasia showing lymphocytic proliferation in subepithelial and deep connective tissue (H&E ×100).

On clinical examination, well-circumscribed red nodules approximately 1 × 1 cm and 1.5 × 1.25 cm in size were noted on the right and left lateral borders of posterior third of the tongue, respectively (Figure 3). The surface was smooth. Upon palpation, they were soft and nontender. Reactive or hamartomatous lesions were expected.

The excisional biopsy from the left side was removed in Oral Surgery Department and submitted for microscopic examination in the Department of Oral and Maxillofacial Pathology, Faculty of Dental Medicine, Cairo University. In macroscopic examination, the specimen was received as two small pieces of 0.5 × 1 and 0.8 × 1 cm in size, reddish in color, and soft in consistency.

Histopathological examination revealed a hyperplastic lymphatic tissue containing aggregations of lymphocytes that form germinal centers in some areas. The lesion was covered by hyperplastic keratinized stratified squamous epithelium (Figure 3). The final diagnosis was designated as a lymphoid hyperplasia.

## 4. Case  3

A ten-year-old female patient came to the Department of Oral and Medicine, Faculty of Dental Medicine, Cairo University, complaining of a swelling and multiple red areas on the dorsal surface of the tongue.

**Figure 4:**
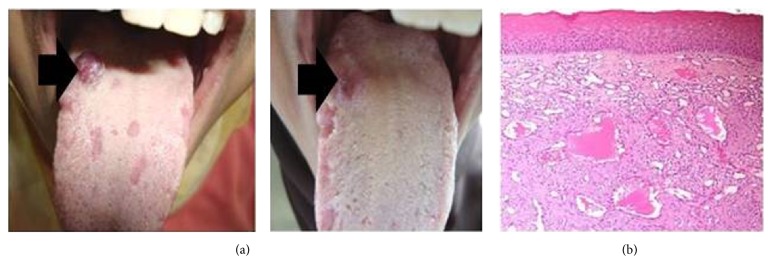
(a) Pre- and postoperative clinical pictures of lymphangioma in relation to the right posterior area of the dorsal surface of the tongue. (b) Photomicrograph of lymphangioma showing numerous subepithelial lymphatic vessels, some of which contain coagulated lymph (H&E ×100).

On clinical examination, well-circumscribed bluish nodule approximately 1.2 × 1.2 cm in size was noted on the right dorsal surface of the tongue (Figure 4). The surface was smooth. Upon palpation, it was soft and nontender. In addition, four depapillated red areas were detected in dorsal and lateral surfaces of tongue. The lingual mass was excised and submitted for pathological examination. In macroscopic examination, the received specimen was as 1 × 1 cm in size, bluish in color, and soft in consistency.

Histopathological examination revealed numerous variably sized lymphatic vessels, some of which contain coagulated lymph. The lesion was covered by keratinized stratified squamous epithelium (Figure 4). The final diagnosis was designated as a lymphangioma.

Five days later, the healing of the area of surgery was reexamined and the disappearance of red areas was noticed (Figure 4).

## 5. Discussion

Swellings in the posterior part of the tongue present a diagnostic and therapeutic dilemma due to their different histogenesis, nature, and subsequently behavior. Slowly growing, painless nonulcerative growths are usually benign while presence of pain, bleeding, ulcer, and induration are characteristic for malignancy. However, some overlapping clinical features are encountered. Therefore, biopsy is usually required to differentiate benign lesions from premalignant and malignant lesions [3, 7, 15].

Oral lymphoepithelial cysts (OLC) are rare lesions arising commonly in the floor of the mouth followed by lateral border and ventral surface of the tongue. Few cases were also reported in soft and hard palate, retromolar area, palatoglossal arch, and palatine tonsil [12, 16, 17].

Despite early description of this type of cysts, the pathogenesis is still debatable. Sethi and Patankar have suggested that oral lymphoepithelial cyst is a pseudocyst caused by obstruction in the crypt of tonsil orifice [16], while Bhaskar identified the ectopic glandular epithelium present within lymphoid tissue of oral mucosa, when undergoing cystic changes, as an origin of lymphoepithelial cyst [8].

The LC can occur in any age with the majority of cases being usually diagnosed in the second and third decade with a slight male predilection [11, 12].

As noticed in our case, clinically, OLC appears as a solitary small soft swelling usually with the color similar to that of adjacent mucosa. However, in some cases, it may appear as yellow papule due to the presence of keratin in its lumen, which leads to a creamy or cheesy appearance.

Histopathologically, the OLC reveals a cystic cavity lined by a stratified squamous epithelium with desquamated keratin in the lumen. The connective tissue wall is usually formed of diffuse lymphoid tissue with frequently observed germinal centers. Our case showed all of these features [12].

Surgical excision is usually done to be examined and exclude the malignant probabilities. Rare recurrence rate was reported with no malignant transformation potentiality [11, 16].

Lymphoid hyperplasia (LH) is an uncommon benign entity related to a rapid proliferation of lymphocytes contained within or outside of lymph nodes. The majority of existing head and neck reports are of hyperplasia in the oral cavity, namely, of the mucosa overlying the hard palate. The exact etiology is not clearly understood, but the reactive nature is strongly suggested [14].

It was called pseudolymphoma due to the great similarity between their clinical and histological pictures [13].

LH affects commonly older female. It may arise as a unilateral, painless, slow-growing, nonulcerated mass. But multifocal lesions were also reported. Histologically, LH is formed of dense lymphoid hyperplasia within the papillary and deep submucosa. Occasionally, germinal centers may also be seen. Absence of cellular monotony and signs of malignancy supports the exclusion of lymphoma [13, 14].

In our case, an old nonsmoker female patient was complaining of slowly growing bilateral swellings in posterior part of lateral borders of the tongue.

Histologically, the examined specimen showed dense lymphoid hyperplasia with few germinal centers covered by hyperplastic stratified squamous epithelium. No signs of malignancy were detected. Both clinical and histological pictures were consistent with previously reported data.

*Lymphangiomas* are benign, hamartomatous proliferations of lymphatic vessels. They most likely represent developmental malformations that arise from sequestrations of lymphatic tissue that do not communicate normally with the rest of the lymphatic system [18, 19].

They appear in the first few years of life, grow slowly, and sometime resolve spontaneously. They were classified according to the size of lymphatic vessels into lymphangioma simplex (capillary lymphangioma), which consists of small lymphatic capillaries, cavernous lymphangioma, which contains dilated lymphatic vessels, and cystic lymphangioma (cystic hygroma), which exhibits large, macroscopic cystic spaces [19].

However, all of these variants of vessels may be found within the same lesion. Like our case, oral lymphangioma occurs frequently on anterior two-thirds of the tongue with superficial location and pebbly surface. However, deeply located lesions were also reported that cause diffuse swelling, termed macroglossia [18, 19].

Histopathological features of our case revealed small capillary sized lymphatic vessels containing proteinaceous fluid and occasional lymphocytes. These vessels were superficially located just beneath the epithelial surface. The most probable type is capillary lymphangioma.

The other clinical finding noticed in the same patient was the presence of red areas that resolved spontaneously. It met the features of geographic tongue.

*Geographic tongue* is also known as benign migratory glossitis or erythema migrans. It is benign self-limiting condition of unknown etiology that is characterized by depapillated red areas and requires no treatment except reassurance. Mild sensitivity to hot or spicy foods was reported [3].

## 6. Conclusion

Histological examination is only safer way to determine the exact nature of the tongue swellings for the selection of appropriate treatment.

## Figures and Tables

**Table 1:** Different types of tongue swellings adapted from Neville et al. [5].

Tongue swelling	Common site	Nature
Squamous cell papilloma	Tip	Benign neoplasm
Verruca vulgaris	Tip	Reactive
Granular cell tumor	Dorsal surface	Benign neoplasm
Sialithiasis	Ventral surface	Reactive
Salivary gland neoplasms	Anterior two-thirds	Benign and malignant lesion
Irritational fibroma	Lateral side	Reactive
Squamous cell carcinoma	Lateral and ventral surfaces	Malignant neoplasm
Lymphoepithelial cyst	Posterolateral and ventral surfaces	Reactive
Lymphoid hyperplasia	Posterolateral side	Reactive
Lymphoma	Posterior third	Malignant neoplasm
Haemangioma	Anterior two-thirds	Developmental
Lymphangioma	Dorsal surface	Developmental
